# The combination of circulating long noncoding RNAs AK001058, INHBA-AS1, MIR4435-2HG, and CEBPA-AS1 fragments in plasma serve as diagnostic markers for gastric cancer

**DOI:** 10.18632/oncotarget.15628

**Published:** 2017-02-22

**Authors:** Dong Ke, Hanwei Li, Yi Zhang, Yinghong An, Hanjiang Fu, Xuedong Fang, Xiaofei Zheng

**Affiliations:** ^1^ General Surgery, The Second Hospital of Jilin University, Changchun, 130041, China; ^2^ Beijing Key Laboratory for Radiobiology, Beijing institute of Radiation Medicine, Beijing, 100850, China; ^3^ Gastrointestinal Colorectal and Anal Surgery, The China-Japan Union Hospital of Jilin University, Changchun, 130033, China; ^4^ Clinical Laboratory Center, Chinese PLA Air Force General Hospital, Beijing, 100142, China

**Keywords:** gastric cancer, long noncoding RNA, plasma, biomarker, RNA fragments

## Abstract

**Background:**

Suitable diagnostic markers for cancers are urgently required in clinical practice. Long non-coding RNAs, which have been reported in many cancer types, are a potential new class of biomarkers for tumor diagnosis.

**Results:**

Five lncRNAs, including AK001058, INHBA-AS1, MIR4435-2HG, UCA1 and CEBPA-AS1 were validated to be increased in gastric cancer tissues. Furthermore, we found that plasma level of these five lncRNAs were significantly higher in gastric cancer patients compared with normal controls. By receiver operating characteristic analysis, we found that the combination of plasma lncRNAs with the area under the curve up to 0.921, including AK001058, INHBA-AS1, MIR4435-2HG, and CEBPA-AS1, is a better indicator of gastric cancer than their individual levels or other lncRNA combinations. Simultaneously, we found that the expression levels of a series of MIR4435-2HG fragments are different in gastric cancer plasma samples, but most of them higher than that in healthy control plasma samples.

**Materials and Methods:**

LncRNA gene expression profiles were analyzed in two pairs of human gastric cancer and adjacent non-tumor tissues by microarray analysis. Nine gastric cancer-associated lncRNAs were selected and assessed by quantitative real-time polymerase chain reaction in gastric tissues, and 5 of them were further analyzed in gastric cancer patients’ plasma.

**Conclusions:**

Our results demonstrate that certain lncRNAs, such as AK001058, INHBA-AS1, MIR4435-2HG, and CEBPA-AS1, are enriched in human gastric cancer tissues and significantly elevated in the plasma of patients with gastric cancer. These findings indicate that the combination of these four lncRNAs might be used as diagnostic or prognostic markers for gastric cancer patients.

## INTRODUCTION

Gastric cancer (GC) is one of the most common malignant tumors [1] and the second most frequent cancer associated with mortality, partially owing to the fact that it is often diagnosed at an advanced stage, and not at the early stage [2, 3]. It is vitally important from a clinical perspective to seek effective tools for the early detection of GC.

Long non-coding RNAs (lncRNAs) are a newly discovered class of ncRNAs longer than 200 nucleotides in length [4]. Changes in lncRNA expression levels have been increasingly reported in a variety of cancer types, suggesting a correlation between lncRNAs and carcinogenesis [5, 6]. Therefore, changes in lncRNA expression levels can serve as a novel target for cancer diagnosis and therapy. For example, lncRNAs can serve as potent biomarkers for the diagnosis and prognosis of clear cell renal cell carcinoma [7]. Overexpression of lncRNA ZFAS1 is associated with intrahepatic and extrahepatic metastasis and poor prognosis of HCC [8]. Moreover, lncRNA HIF1A-AS1, regulates proliferation and migration of oesophageal adenocarcinoma cells [9]. Level of H19 was higher in GC patient plasma than healthy controls with the area under curve(AUC) up to 0.838 [10]. However, Some lncRNAs, highly expressed in GC tissues, served as markers with low AUCs [11, 12]. Given that single lncRNA management, the AUC is too low to discriminate GC patients from healthy controls. Therefore, it is important to discover lncRNAs with higher sensitivity and specificity that can be used as a biomarker for GC diagnosis.

In this study, nine lncRNAs, dysregulated in GC tissues identified by lncRNA microarray, were selected to evaluate their expression in gastric tissues and plasma samples. The purpose of our study was to determine whether these lncRNAs could discriminate GC patients from healthy controls. In addition, we analyzed the expression characteristics of diverse lncRNA fragments in plasma samples, and the potential relationship between circulating lncRNA levels and the clinicopathological features of GC.

## RESULTS

### Expression profile of lncRNAs in gastric cancer

LncRNA gene expression profiles were analyzed in two pairs of human GC and adjacent non-tumor (NT) tissues by microarray analysis. Fold-change (Tumor vs. Normal) and *P* value were calculated from the normalized expression (Fold-change ≥ 2 or ≤ 0.5, *p* < 0.05). The microarray data has been deposited in NCBI Gene Expression Omnibus (GEO) and the GEO accession number is GSE93512. In total, 154 lncRNAs were identified to be consistently increased (Supplementary Figure 1A) in all two GC groups, and 238 lncRNAs were consistently decreased (Supplementary Figure 1B). Among these, 9 lncRNAs, showing significant difference in both tissue microarrays, were selected for further validation (Supplementary Table 1). Of these 9 lncRNAs, INHBA-AS1, MIR4435-2HG, UCA1, AK001058, LOC100133091, and MGC12916 were increased, where as CEBPA-AS1, FLJ37453, and LINC01184 were decreased in GC tissues.

### Five lncRNAs were increased in GC tissues

**Figure 1:**
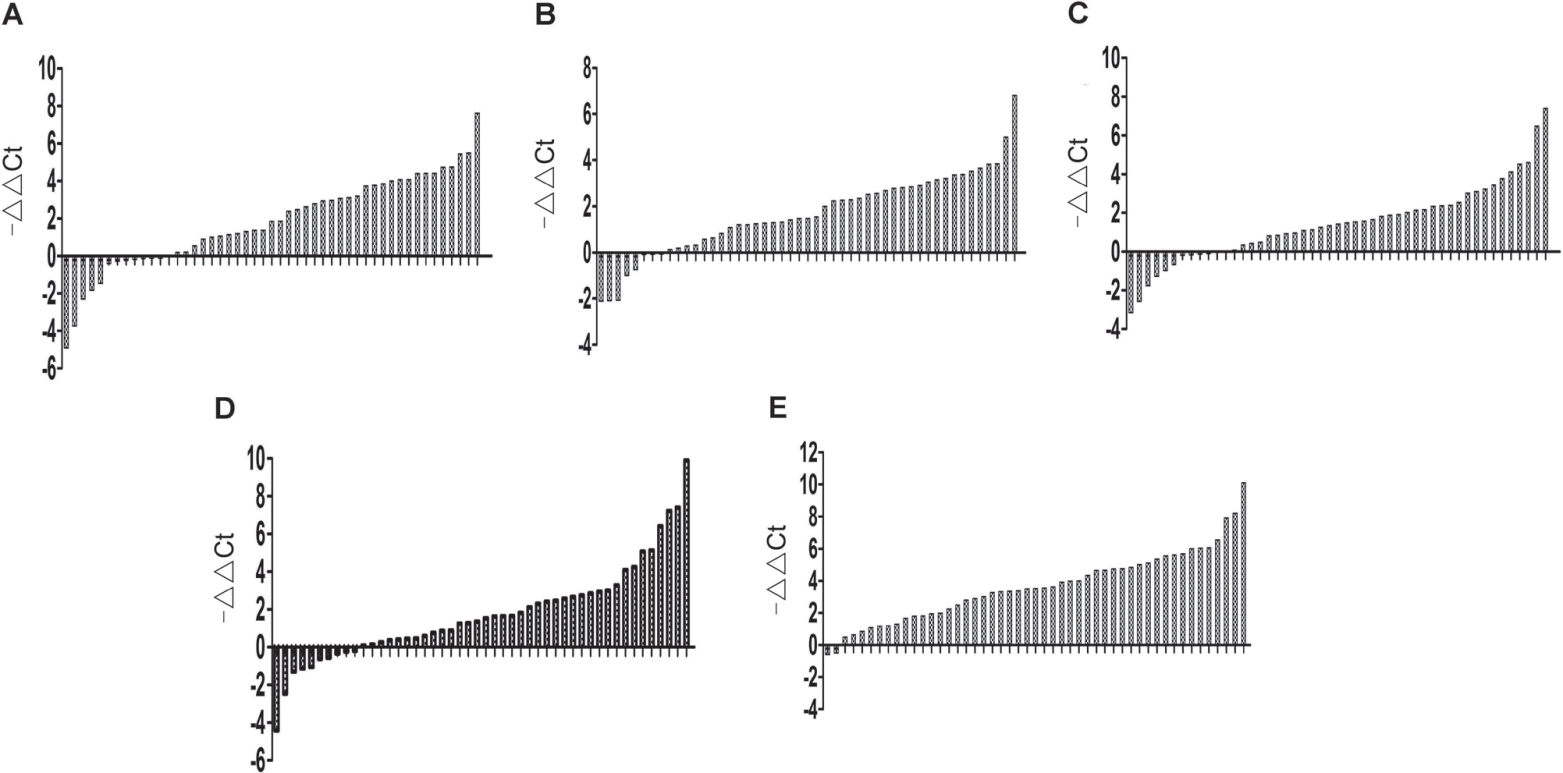
Gene expression levels in gastric tissues Expression level of lncRNA INHBA-AS1 (**A**), MIR4435-2HG (**B**), CEBPA-AS1 (**C**), UCA1 (**D**), and AK001058 (**E**) in 49 paired gastric tissues. -ΔΔCt= (Ct_target gene in_ _normal_-Ct_18S rRNA in normal_) - (Ct_target gene in_ _cancer_-Ct_18S rRNA in cancer_).

Based on the gastric tissue microarray results, we validated the expression of the 9 lncRNAs in 49 GC tissues and adjacent NT tissues using qRT-PCR. Selection of an appropriate reference gene is crucial to the analysis. RNA expression was normalized to that of β-actin [13, 14] or 18S rRNA as described previously [15, 16]. In this study, 18S rRNA was selected as the reference gene, because the expression level of 18S rRNA was not significantly different between GC tissues and adjacent NT tissues. We first examined 18 paired gastric tissues, but of the 9 selected lncRNAs, lncRNA FLJ37453, LINC01184, LOC100133091, and MGC12916 did not show marked changes (results not shown). Next, we examined the other five lncRNAs in the remaining 31 paired gastric tissues. LncRNAs INHBA-AS1, MIR4435-2HG, CEBPA-AS1, UCA1, and Ak001058 were increased in 37 (75.51%), 41 (83.67%), 39 (75.59%), 39 (75.59%), and 47 (95.92%) of the 49 GC tissues, respectively (Figure 1A–1E). The relationship between lncRNA levels in tissues and the clinicopathological features of GC patients was also analyzed (Table 1). The expression levels of INHBA-AS1, MIR4435-2HG, CEBPA-AS1, and AK00108 were associated with tumor grade (Supplementary Figure 2A–2D); AK001058 had a higher expression level in GC tissues with lymph node metastasis compared to that with no lymph node metastasis (Supplementary Figure 2E), and the expression level of UCA1 was higher in GC I stage than that in GC II-IV stage (Supplementary Figure 2F). The AUCs for INHBA-AS1, MIR4435-2HG, CEBPA-AS1, UCA1, and AK001058 were 0.740, 0.770, 0.741, 0.722, and 0.957, respectively (Supplementary Figure 3A). The AUC value of the combination of 5-lncRNA was up to 0.976 (95%CI: 0.000–1.000) (Supplementary Figure 3B), when the AUC value of a single lncRNA was lower than that of the 5-lncRNA signature.

**Table 1:** Correlation between lncRNA-INHBA-AS1, MIR4435-2HG, CEBPA-AS1, UCA1, and AK001058 panel expression levels in gastric tissues and clinical parameters

Clinical parameters	Number of cases	*p*-value				
		INHBA-AS1	MIR4435-2HG	CEBPA-AS1	UCA1	AK001058
**Age(years)**						
< 60	19	0.2067	0.0251 (*)	0.8578	0.0571	0.2048
> 60	30					
**Gender**						
female	18	0.2906	0.0589	0.2672	0.8692	0.5503
male	31					
**Tumor grade**						
Low	28	0.0114(*)	0.0416(*)	0.0313(*)	0.4828	0.0330(*)
middle or high	21					
**LN metastasis**						
No	17	0.9298	0.4592	0.8496	0.2886	0.0350(*)
Yes	32					
**Depth of invasion**						
T1-2	11	0.7742	0.6702	0.2251	0.5407	0.7243
T3-4	38					
**TNM stage**						
I	19	0.7255	0.7011	0.7996	0.0185(*)	0.2222
II-IV	30					

Abbreviations: lncRNA, long noncoding RNA; LN, lymph node. TNM, tumor node metastasis; *Statistically Significant (*p* < 0.05).

### Correlation of antisene lncRNAs expression and their corresponding mRNAs expression in gastric cancer tissues

Most protein coding genes (PCGs) have their associated antisense RNA, which can interact with nearby associated PCGs. LncRNAs are reportedly able to regulate all steps of the gene expression process [17]. Numerous studies have focused on the analysis of the expression patterns of lncRNAs and their possible crosstalk with adjacent protein-coding genes. The antisense lncRNA Khps1 activates SPHK1 transcription by targeting chromatin modifying enzymes to the SPHK1 promoter and changing chromatin structures [18]. RBM15-AS1, transcribed in the opposite direction within exon 1 of RBM15 was increased in megakaryocyte and activated megakaryocyte differentiation and may play a regulatory role in leukemogenesis by enhancing RBM15 protein translation[19]. INHBA-AS1 and CEBPA-AS1 are the antisense RNAs of INHBA and CEBPA, respectively. CEBPA-AS1 and CEBPA were both increased in 23 (95.8%) and decreased in 1 (4.17%) GC tissues (Supplementary Figure 4A). INHBA-AS1 and INHBA were both increased in 19 (78.2%) (Supplementary Figure 4B) among 24 paired GC tissues. According these results, we found that the changing trend of CEBPA, INHBA, and their antisense RNA basically identical. It would be valuable to study the functional relationship between INHBA-AS1, CEBPA-AS1 and their related PCGs.

### Detection of plasma expression of the 5 lncRNAs fragments in the training set

**Figure 2:**
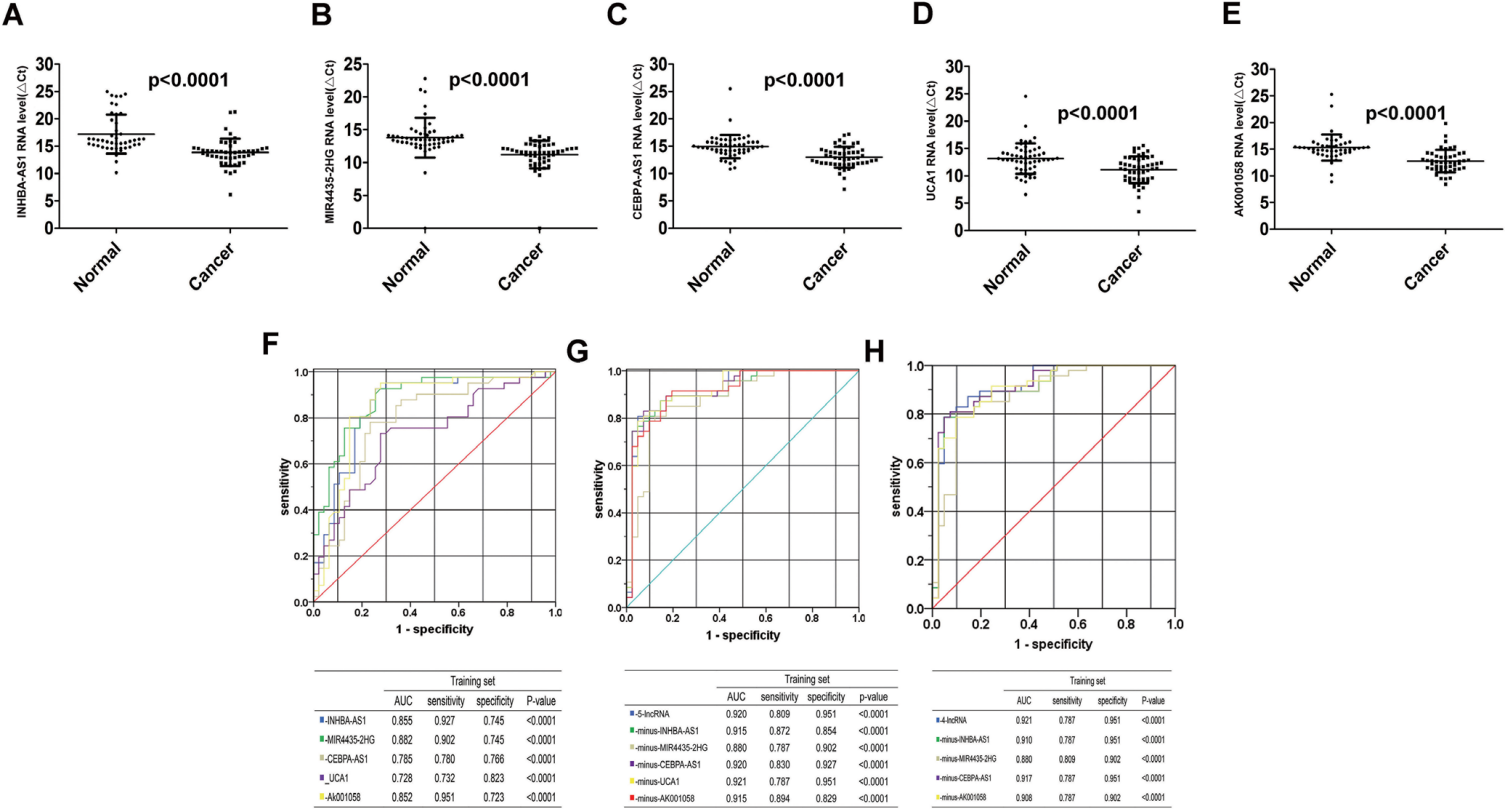
Distribution of lncRNAs in plasma and AUC for lncRNA panels in training set Distribution of lncRNA-INHBA-AS1 (**A**), MIR4435-2HG (**B**), CEBPA-AS1 (**C**), UCA1 (**D**), and AK001058 (**E**) levels in the plasma from patients and healthy controls in the training set. ΔCt_target gene in_ _cancer_= Ct_target gene in_ _cancer_-Ct_18S rRNA in cancer_; ΔCt_target gene in_ _normal_= Ct_target gene in_ _normal_-Ct_18S rRNA in normal_. And the ROCs of the 5 lncRNA (**F**), 5-lncRNA and five-minus-one lncRNA (**G**), and a new 4-lncRNA signature and four-minus-one lncRNA (**H**) in training set expressed in plasma, respectively. The AUCs and *p*-values are listed in the picture.

**Figure 3:**
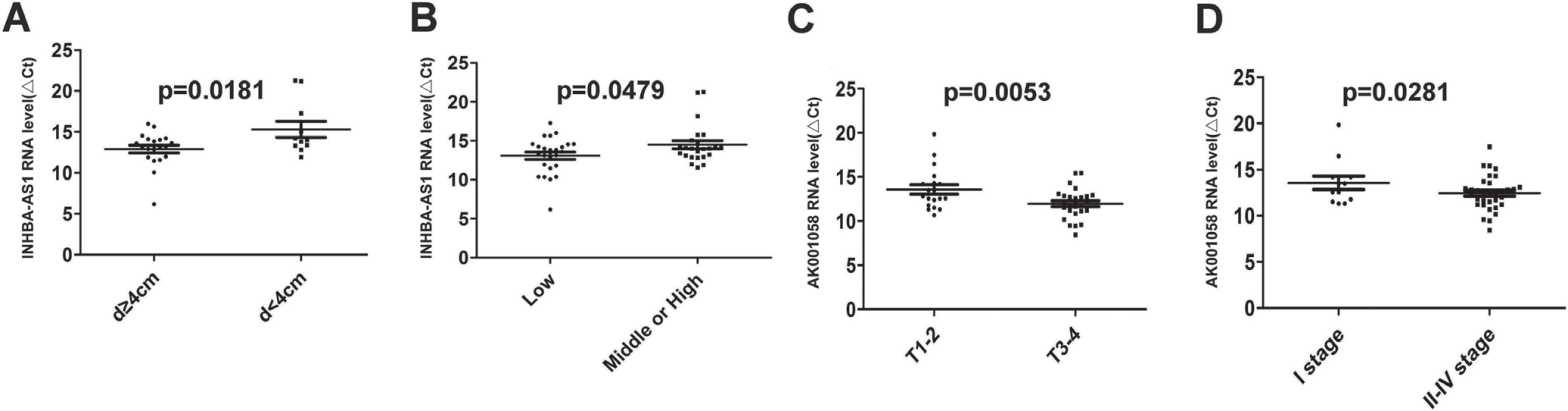
The relationship between lncRNA levels and the clinicopathological features of GC patients in training set The relationship between the expression level of INHBA-AS1 and tumor size (**A**) and tumor grade (**B**); the relationship between the expression level of AK001058 and the depth of tumor invasion (**C**) and TNM stage (**D**). *p*-values are listed in the picture. ΔCt_target gene in_ _cancer_= Ct_target gene in_ _cancer_-Ct_18S rRNA in cancer_.

We validated the 5 lncRNAs that were increased in the GC tissues in 51 GC patients and 53 healthy controls, the plasma samples were collected from Air Force General Hospital, PLA, Beijing, China. All 5 lncRNAs, including INHBA-AS1, MIR4435-2HG, CEBPA-AS1, UCA1 and AK001058 were increased in the GC plasma (Figure 2A–2C, 2E), exhibiting the same trend as in the GC tissues. With an AUC of 0.855, 0.882, 0.785, 0.728, and 0.852 for INHBA-AS1, MIR4435-2HG, CEBPA-AS1, UCA1, and AK001058, respectively (Figure 2F), the AUC for UCA1 was lowest in the five lncRNAs. Furthermore, the AUC of the 5-lncRNA panel was 0.920 (95%CI: 0.860–0.981) (Figure 2G), when the AUC of a single lncRNA was lower than that of the 5-lncRNA signature. And a five-minus-one lncRNA signature was constructed, the five-minus-UCA1 lncRNA signature has a higher AUC (AUC = 0.921; 95%CI: 0.862–0.981) than the others and the 5-lncRNA signature (Figure 2G). We also constructed a 3-lncRNA signature (Figure 2H, Supplementary Table 2) and 2-lncRNA signature (Supplementary Table 3), randomly chose from the 5 lncRNAs, their AUCs were also lower than the five-minus-UCA1 lncRNA signature. So a new 4-lncRNA signature, including INHBA-AS1, MIR4435-2HG, CEBPA-AS1, and AK001058, was constructed. The relationship between lncRNA levels in the plasma and the clinicopathological features of GC is shown in Table 2. The expression level of INHBA-AS1 was associated with tumor size (Figure 3A) and tumor grade (Figure 3B); There is a correlation between the expression level of AK001058 and depth of tumor invasion (Figure 3C) and TNM stage (Figure 3D).

**Table 2:** Correlation between lncRNA-INHBA-AS1, MIR4435-2HG, CEBPA-AS1, UCA1, and AK001058 panel expression levels in GC plasma and clinical parameters

Clinical parameters	Number of cases	*p*-value				
		INHBA-AS1	MIR4435-2HG	CEBPA-AS1	UCA1	AK001058
**Age(years)**						
< 60	23	0.0380(*)	0.5922	0.0764	0.1033	0.6995
> 60	28					
**Gender**						
female	16	0.3604	0.4214	0.3135	0.1274	0.5364
male	35					
**Tumor size(cm)**						
≥ 4	21	0.0181(*)	0.8777	0.7252	0.9587	0.8750
< 4	12					
**Tumor grade**						
Low	26	0.0479(*)	0.6296	0.3396	0.5124	0.1324
middle or high	25					
**LN metastasis**						
No	14	0.2922	0.4839	0.2764	0.2712	0.1322
Yes	29					
**Depth of invasion**						
T1-2	21	0.0599	0.0556	0.0614	0.0542	0.0053(**)
T3-4	27					
**TNM stage**						
I	18	0.3756	0.4137	0.5555	0.6802	0.0281(*)
II-IV	30					

Abbreviations: lncRNA, long noncoding RNA; LN, lymph node; TNM, tumor node metastasis; *Statistically Significant (*p* < 0.05).

### Validation of the utility of candidate lncRNAs in the testing set

**Figure 4:**
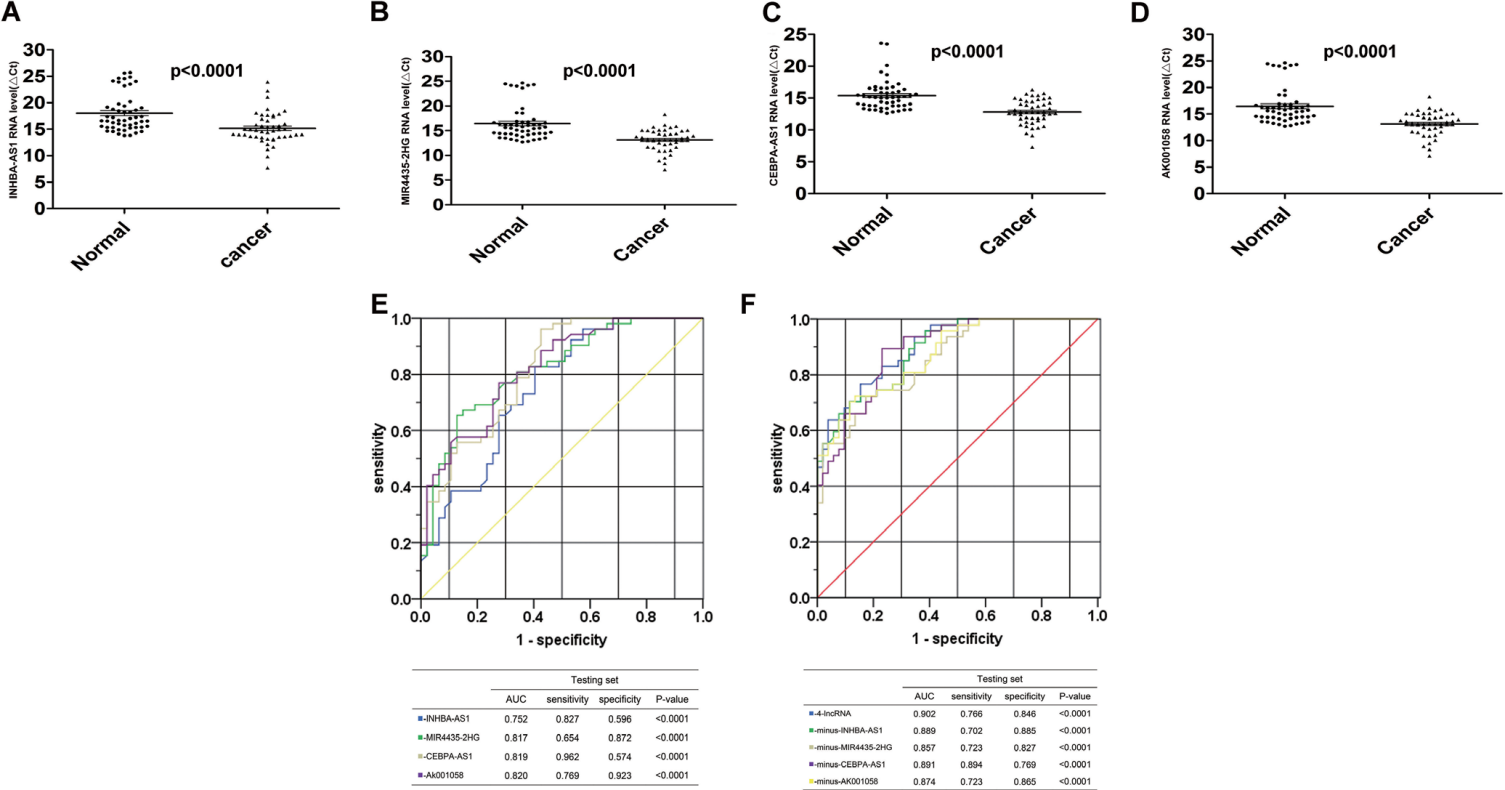
Distribution of lncRNAs in plasma and AUC for lncRNA panels in testing set Distribution of lncRNA-INHBA-AS1 (**A**), MIR4435-2HG (**B**), CEBPA-AS1 (**C**) and AK001058 (**D**) levels in the plasma from patients and healthy controls in the testing set. ΔCt_target gene in_ _cancer_= Ct_target gene in_ _cancer_-Ct_18S rRNA in cancer_; ΔCt_target gene in_ _normal_= Ct_target gene in_ _normal_-Ct_18S rRNA in normal_. The ROCs of the 4 lncRNA (**E**), 4-lncRNA and four-minus-one lncRNA (**F**) expressed in plasma in testing set. The AUCs and *p*-values are listed in the picture.

We validated the 4-lncRNA panel using the same method in a test cohort of 47 GC patients and 52 healthy controls, the plasma samples were collected from the China-Japan Union Hospital of Jilin University, Changchun, China. In the testing set, all the four lncRNAs, including INHBA-AS1, MIR4435-2HG, CEBPA-AS1, and AK001058 were also increased in the plasma samples from GC patients (Figure 4A–4D), and the predictor was also remarkably stable, with AUCs of 0.752, 0.817, 0.819, and 0.820 for INHBA-AS1, MIR4435-2HG, CEBPA-AS1, and AK001058, respectively (Figure 4E). In addition, the AUC value of the 4-lncRNA panel was 0.902 (Figure 4F). Additionally, we constructed 4-minus-one lncRNA signatures by excluding each lncRNA from the set individually and comparing the AUCs of these three lncRNA signatures with that of the original 4-lncRNA signature. None of the four-minus-one lncRNA signatures had a higher AUC value in the training set (Figure 2H) and testing sets (Figure 4F) than those of the 4-lncRNA signature. The AUCs of a 2-lncRNA signature, randomly chose from the 4 lncRNAs were also lower than the 4-lncRNA signature (Supplementary Table 4) in testing sets.

### The expression level of each lncRNA fragment is different in plasma

**Figure 5:**
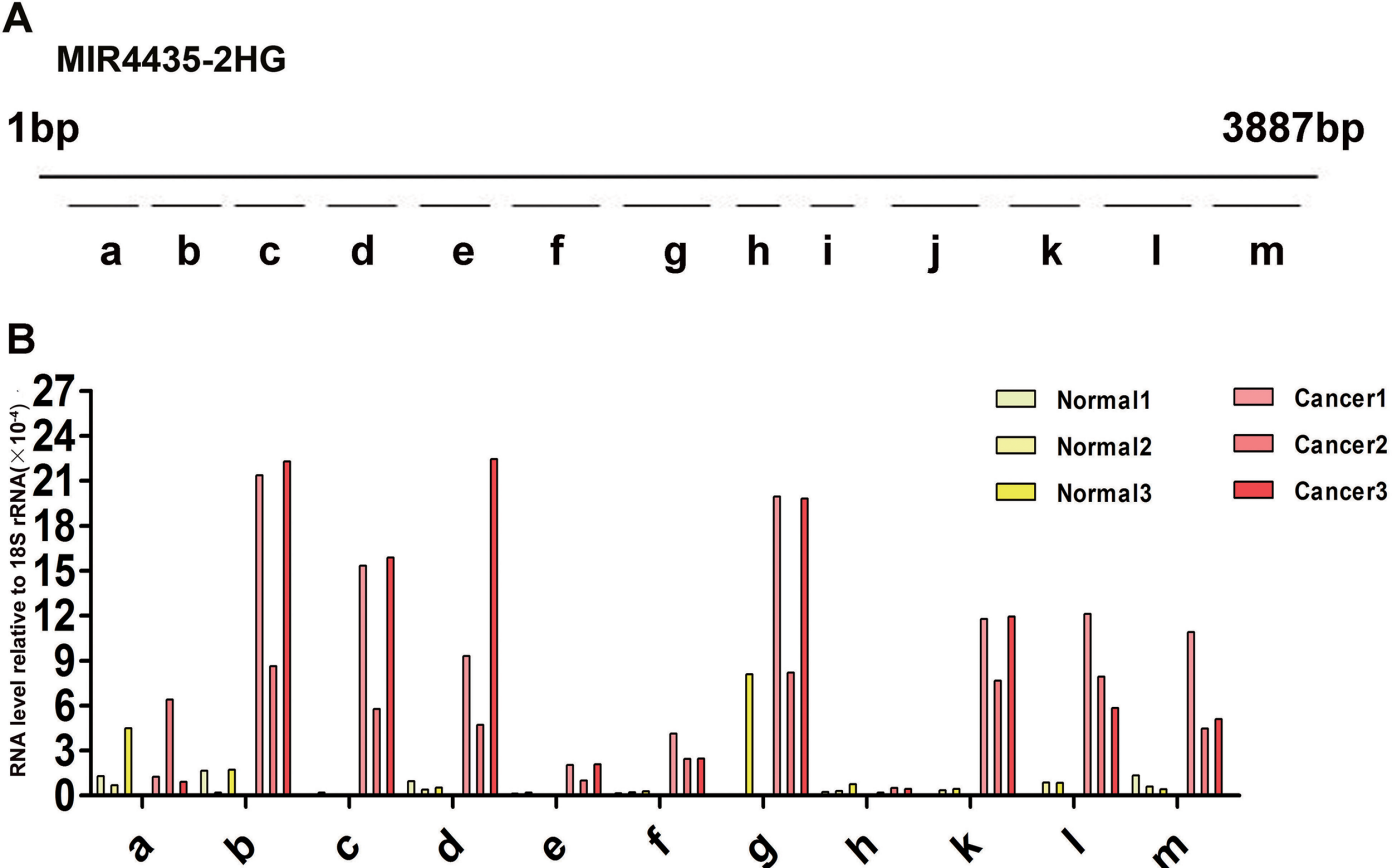
Expression of diverse MIR4435-2HG fragments in health control and gastric cancer plasma (**A**) 13 different primer pairs of MIR4435-2HG gene, a-m fragments are the qRT-PCR products with different length. a: 186–291(106 bp); b: 424–529(106 bp); c: 728–833(106 bp); d: 1035–1151(117 bp); e: 1257–1356(100 bp);f: 1530–166(136bp); g: 1955–2079(125 bp); h: 2270-2359(90 bp); i: 2609–2692(84 bp); j: 2716–2841(126 bp); k: 3164–3273(110 bp); l: 3319-3457(139bp); m: 3657–3791(135 bp). (**B**) Expression level of MIR4435-2HG fragments in three GC patient and health control samples relative to 18S rRNA. Fragment i, j cannot be detected among the six plasma samples, and fragment l can be detected excluding one sample of three healthy controls.

Different from miRNAs, complete lncRNA molecule in plasma were little. Plasma lncRNAs exist mainly in the form of RNA fragments. The contents of lncRNA fragments in plasma might be different from each other. Therefore, it is necessary to evaluate the expression level of different RNA fragments from the same lncRNA in plasma to employ lncRNAs as biomarkers. LncRNA MIR4435-2HG, which was increased in GC plasma, was selected for the following test. MIR4435-2HG is a lncRNA with 3887bp, we designed 13 pairs of primers to detect the expression levels of different RNA fragments from MIR4435-2HG (Figure 5A, Supplementary Table 5) by qRT-PCR. Each of fragments is ∼300bp, among these possible RNA fragments, i and j were not detected in all GC and healthy control plasma samples. Fragment a could be detected, but its expression level in GC plasma is not significantly different from that in healthy control samples. The levels of fragment b, c, d, g, k, l, m in GC plasma samples are much higher than those in healthy controls (Figure 5B). According to the results, fragment b, c, d, g, k, l or m may be more potential to serve as a biomarker. These data suggest that it is necessary to identify the most stable and differential expressed RNA fragment as diagnostic and prognostic biomarker.

## DISCUSSION

Gastroscopy [20] and computed tomography (PET/CT) [21] are the commonly used tools for the diagnosis of GC. Carcinoembryonic antigen (CEA), CA19-9 [22], and CA-724 [23] are classic tumor markers commonly used in the management of GC [24]. Given that these markers with low diagnostic sensitivity and specificity have limited utility [25], discovery of new biomarkers has attracted attention for early detection of GC.

An increasing number of studies have demonstrated that circulating nucleic acids (CNAs) can be found in cell-free plasma, serum, and other body fluids. For example, miRNAs are differentially expressed in various types of cancers and possess good diagnostic value in cancer screening [26–28]. In some cases, circulating cell-free DNAs (cfDNAs) are also dysregulated in the plasma and serum of cancer patients [29, 30]. In non-small cell lung cancer, cfDNA could be served as a prognostic and predictive biomarker [31].

Given that plasma is relatively easy to obtain and genome-wide screening approaches provide us new opportunities to develop novel diagnostic or prognostic markers, blood based testing is ideal for evaluating biomarkers in cancer. Some lncRNAs or miRNAs that stably exist in plasma may be protected by exosome encapsulation, microvesicles, apoptotic bodies [32, 33] and complex formation with proteins [34]. Therefore, lncRNAs can serve as promising markers for early diagnosis of cancers.

In our study, we systematically determined the expression levels of nine cancer-associated lncRNAs dysregulated in two gastric tissue microarrays and detected their relative expression levels in paired gastric tissues by qRT-PCR. Expression of CEBPA protein was increased in HCCs [35], and epigenetic aberrations in regulating CEBPA expression contributed to leukemic transformation in acute myeloid leukemia[36]. INHBA mRNA and protein expression is commonly elevated in primary human NSCLC, and it promotes tumor metastasis [37]. Here, we found that there is a positive relation between the RNA levels of INHBA-AS1 and CEBPA-AS1 with their PCGs in GC tissues, respectively (Supplementary Figure 4A, 4B). These two lncRNAs might influence the expression of their nearby PCGs through certain mechanism. Interestingly, CEBPA-AS1 was shown to be decreased in two GC tissues by microarray analysis, it is increased in most GC tissues in fact (Figure 1C). That maybe be because it is decreased in the two GC tissues by chance. Furthermore, Ak001058 is increased in 47 (95.92%) of the 49 GC tissues (Figure 1E), and the AUC value for AK001058 is 0.957 (Supplementary Figure 3A). These results suggest that Ak001058 is very sensitive, might be developed into a novel biomarker for diagnosis of GC.

With lower AUC value, UCA1 expression level was not changed remarkably in GC plasma. Notably, we established a 4-lncRNA signature not including UCA1, and our results showed that the 4-lncRNA panel was highly indicative of GC diagnosis. The AUC values of this 4-lncRNA panel were 0.921 and 0.902 in the training and testing sets, respectively, which was higher than that of any panels (Figures 2, 4). Among the four lncRNAs, the RNA level of AK001058 was higher in GC advanced stage (Figure 3), suggesting that this gene can predict GC at a relatively early stage.

LncRNA MIR4435-2HG is related to cell-cycle in lung cancer cells [38]. Here, we found that MIR4435-2HG was also increased in GC tissues. Interestingly, our results demonstrate that different fragments of MIR4435-2HG have different expression levels, and the level of the same fragment was different in GC plasma and healthy controls. The primer of MIR4435-2HG in this study is complementary with fragment b. All these data claims that lncRNAs exist mainly in the form of RNA fragments in plasma. Therefore, it is necessary to identify the most stable and differential expressed RNA fragment as diagnostic biomarker.

Taken together, the results show that the plasma 4-lncRNA panel constructed could not only discriminate GC patients from healthy controls, but also displayed potential use for diagnosing GC at an early stage. Not all, but some specific fragments of lncRNAs in plasma could be used as an appropriate biomarker. Our work may facilitate the detection of GC and serve as the basis for further studies of plasma lncRNAs in predicting personalized treatment strategies and efficacy of GC patients.

## MATERIALS AND METHODS

### Patients and clinical samples

A total of 49 primary cancer tissues and the paired adjacent non-tumor tissue were collected from patients who underwent surgery for GC at the China-Japan Union Hospital of Jilin University. These tissues were flash frozen in liquid nitrogen immediately after surgery and subsequently stored at −80°C. The plasma samples were collected from the China-Japan Union Hospital of Jilin University and Air Force General Hospital, PLA. All plasma samples were stored at −80°C. No patients received anticancer treatments before surgery in this study. All samples were staged in accordance with the tumor node metastasis (TNM) classification and criteria of the Union for International Cancer Control (UICC), and tumor grade was assessed according to the UICC criteria. Written informed consent was obtained from all patients(Supplementary Tables 6, 7, 8). The Ethics Committee of the China-Japan Union Hospital of Jilin University and Air Force General Hospital, PLA approved the use of samples for this study.

### Sample preparation and tissue microarray

Two groups of gastric tissue samples, including GC tissue and adjacent non-tumor tissue, were prepared. RNA was extracted from the frozen tissue using TRI reagent (Sigma, USA), RNA integrity was analyzed by denatured agarose gel electrophoresis. Microarray hybridization was performed by Kangchen Biotech, Shanghai P. R. China.

### Tissue and plasma RNA extraction

Total tissue RNA was extracted from the frozen tissue block using TRI reagent (Sigma, USA) according to the manufacturer's protocol. Plasma cell-free RNA was extracted from 200 μL of plasma using the miRNeasy Serum/Plasma Kit (QIAGEN, German) according to the manufacture's protocol. All RNA and cDNA products were stored at −80°C until use.

### Reverse transcription (RT) and quantitative real-time polymerase chain reaction (qRT-PCR) validation

Total RNA was reverse-transcribed using ImProm II Reverse Transcriptase (Promega, USA) according to the manufacturer's instructions. qRT-PCR was performed with SYBR Premix ExTaq (TaKaRa, Japan) on an MX3000p instrument (Agilent Technologies, USA) according to the manufacturer's protocol. For tissues, RT products were diluted for 100 times, and the dilutions were used as new templates to detect the expression level of 18S rRNA. Amplification of the appropriate product was confirmed by a melting curve analysis following amplification. The relative expression of each lncRNA was calculated using comparative cycle threshold (CT) (2^−ΔCt^) method with 18S rRNA as the endogenous control for data normalization. The method of analyzing relative gene expression is described in detail in this paper [39].

### Statistical analysis

The statistical analysis was performed using the SPSS 17.0 and GraphPad Prism 5.0 softwares. Student's *t*-test was used to evaluate differences in the expression of the chosen lncRNAs in tissues and plasmas from the GC patients and healthy controls. The specificity, sensitivity, and AUC for the lncRNA levels were determined using an ROC analysis. Using the binary outcome of GC and healthy control samples as dependent variables, a logistic regression model was established using the stepwise model selection method. All of the statistical tests were two tailed, and *P* value < 0.05 was considered statistically significant.

## CONCLUSIONS

One of the major strengths of this study is that we found that the combination of plasma lncRNAs is a better indicator of GC, and these four lncRNAs might be used as diagnostic or prognostic markers and therapeutic targets for GC patients.

We also identified that the expression levels of a series of MIR4435-2HG fragments are different in GC plasma samples, these results imply that the expression level of different RNA fragments from the same lncRNA in plasma to employ lncRNAs as biomarkers.

## SUPPLEMENTARY MATERIALS FIGURES AND TABLES



## Abbreviations

GC: gastric cancer
AUC: Area under the curves
Cl: Confidence interval
ROC: Receiver operating characteristic
lncRNAs: long noncoding RNAs
RT: Reverse transcription
TNM: tumor node metastasis
qRT-PCR: quantitative real-time polymerase chain reaction
NT: non-tumor
UICC: the Union for International Cancer Control
Ct: cycle threshold
miRNAs: microRNAs
CNAs: circulating nucleic acids
cfDNAs: circulating cell-free DNAs
UCA1: urothelial cancer associated 1
CEBPA: CCAAT/enhancer binding protein alpha
CEBPA-AS1: CCAAT/enhancer binding protein alpha antisense RNA 1
INHBA: inhibin beta A subunit
INHBA-AS1: inhibin beta A subunit antisense RNA1
MIR4435-2HG: microRNA 4435-2 host gene
