# Supplementary material for: The combination of circulating long noncoding RNAs AK001058, INHBA-AS1, MIR4435-2HG, and CEBPA-AS1 fragments in plasma serve as diagnostic markers for gastric cancer
Source: Oncotarget. 2017 Feb 22;8(13):21516–25. doi: 10.18632/oncotarget.15628 (PMC5400602; doi:10.18632/oncotarget.15628)
Supplement: Supplementary file 1 [file oncotarget-08-21516-s001.pdf]

The combination of circulating long noncoding RNAs AK001058, INHBA-AS1, MIR4435-2HG, and CEBPA-AS1 fragments in plasma serve as diagnostic markers for gastric cancer

Supplementary Materials

Supplementary Table 1: The information for selected 9 lncRNA

gene name	seqname	chromosome	ncRNA type	antisense RNA
AK001058	AK001058	chr5	intergenic	
INHBA-AS1	NR_027119	chr7	intronic antisense	INHBA
MIR4435-2HG	NR_015395	chr2	intergenic	
LINC01184	NR_024125	Chr1	nature anstisense	
MGC12916	NR_026880	chr17	intron sense-overlapping	HS3ST3B1
CEBPA-AS1	NR_026887	chr19	bidirectional	CEBPA
UCA1	NR_015379	chr19	intergenic	
FLJ37453	NR_024279	chr1	natural antisense	POMZP3
LOC100133091	NR_029411	chr7	natural antisense	

Supplementary Table 2: The area under the curve of 3-lncRNA signature randomly chose from 5 lncRNAs in training set

	AUC	sensitivity	specificity	*p*-value
I+M+U	0.898	0.843	0.868	< 0.0001
I+C+U	0.833	0.765	0.849	< 0.0001
I+U+A	0.828	0.706	0.868	< 0.0001
M+C+U	0.869	0.784	0.906	< 0.0001
M+U+A	0.892	0.824	0.925	< 0.0001
C+U+A	0.814	0.843	0.736	< 0.0001

Abbreviations: I, INHBA-AS1; M, MIR4435-2HG; C, CEBPA-AS1; U, UCA1; A, AK001058; lncRNA, long noncoding RNA.

Supplementary Table 3: The area under the curve of 2-lncRNA signature randomly chose from 5 lncRNAs in training set

	AUC	sensitivity	specificity	*p*-value
I+M	0.903	0.702	0.976	< 0.0001
I+C	0.838	0.745	0.878	< 0.0001
I+U	0.836	0.787	0.854	< 0.0001
I+A	0.864	0.766	0.902	< 0.0001
M+C	0.885	0.745	0.902	< 0.0001
M+U	0.897	0.894	0.829	< 0.0001
M+A	0.898	0.723	0.976	< 0.0001
C+U	0.794	0.745	0.805	< 0.0001
C+A	0.852	0.809	0.902	< 0.0001
U+A	0.844	0.809	0.872	< 0.0001

Abbreviations: I, INHBA-AS1; M, MIR4435-2HG; C, CEBPA-AS1; U, UCA1; A, AK001058; lncRNA, long noncoding RNA.

Supplementary Table 4: The area under the curve of 2-lncRNA randomly chose from 4 lncRNAs in testing set

	AUC	sensitivity	specificity	*p*-value
I+M	0.829	0.915	0.596	< 0.0001
I+C	0.832	0.596	0.904	< 0.0001
I+A	0.828	0.809	0.673	< 0.0001
M+C	0.868	0.957	0.615	< 0.0001
M+A	0.880	0.915	0.731	< 0.0001
C+A	0.844	0.723	0.808	< 0.0001

Abbreviations: I, INHBA-AS1; M, MIR4435-2HG; C, CEBPA-AS1; A, AK001058; lncRNA, long noncoding RNA.

Supplementary Table 5: The primers of genes used in the paper

Gene names	Forward (5′-3′)	Reverse (5′-3′)
18S rRNA	GTAACCCGTTGAACCCCATT	CCATCCAATCGGTAGTAGCG
INHBA-AS1	CCTACTACACACAGGGGCTC	TTCCAGAAGCTCCTCATGGG
MIR4435-2HG	GTGTAGGAGAGTCGGCCTTC	TTGGGCTGGGATAGTGTCT
CEBPA-AS1	TGCGTCCCTCGCATTCTTTA	GACAGGAGACACTTGAGGGC
UCA1	AACCATCAGATCCTTGCCCA	AATATGTGGAACTGGCCCCA
AK001058	CTGCTTTGCCATTTCCCCTT	GTTGATGCCACACAGAGGGA
LOC100133091	AGAGTCACTGGGCTACACAC	CCATCAGGCCCAGTATTCCT
LINC01184	GAGAAGTGCAGTTGAGTCGC	CCTTACCCTTCCACTGAGCA
FLJ37453	CTTGCAAGCCCTTCGAAAGT	TTCCCACGGCTGAGTTCTAG
MGC12916	AGCCATCTCCCTGAACTGAC	AGTTGCGGCATTTACAGTGG
MIR4435-2HG -a	GACATTCCAGACAAGCGGTG	TCCACTTTGCTTGTCAGGGA
MIR4435-2HG -b	GTGTAGGAGAGTCGGCCTTC	TTGGGCTGGGATAGTGTCT
MIR4435-2HG -c	ACCCTGTGAGCATGATTGGA	CTCAAAAGACCCAGATGCCG
MIR4435-2HG -d	ACTCTGAAACTACCCGGCTC	GTCAACGCGGAAAAGACACT
MIR4435-2HG -e	TGTCACAACACAACGCTGAG	CTCGCGGTGAACTTGGCC
MIR4435-2HG -f	TAGTGTCTTGCTGGCCTGAA	GCTTAGGAGGGAGAGACACC
MIR4435-2HG -g	TCCAAGAGAGCTGGACAGAC	TTAAGCAATCTGGGGTGGCT
MIR4435-2HG -h	CATCCTCGACAGTGCTTCCT	TTTAGAGGAGCAGAGCAGGG
MIR4435-2HG -i	GAGTGCCACCTATGACCTCA	TGTAGACATCCTCAGGCTCG
MIR4435-2HG -j	GCAGAAGGTGGGGAGGATAA	GGCCCCTTCATAGCTCAGAA
MIR4435-2HG -k	TGAACCTAGGTCAGTCTGGC	ACTGGTGGCTACAAAGGTGA
MIR4435-2HG -l	TTCAGCTGCAAGACGTATGC	TTCCTGACATCATGGAGGCC
MIR4435-2HG -m	AGCCCAGTCAAGAGAGGATG	CCTCACTCCGCTTCTCTAGT

Supplementary Table 6: The clinical parameters of GC patients and tumors

sample	Gender	Age	Tumor grade	Depth of invasion	LN metastasis	TNM stage
1	male	71	low	T3	N3b	IV
2	female	72	low	T3	N1	III
3	male	73	Middle	T3	N3	IV
4	female	47	low	T2	N0	IA
5	male	53	Middle	T2	N0	I
6	male	68	Middle	T3	N3	IV
7	female	57	low	T2	N0	IB
8	female	65	low	T2	N0	IB
9	male	58	Middle	T4	N1	IV
10	male	62	low	T3	N2	IV
11	male	59	low	T3	N3b	IV
12	male	57	low	T3	N3a	IV
13	female	61	low	T1	N0	I
14	female	57	low	T4	N3b	IV
15	male	54	low	T4a	N3a	IV
16	male	86	Middle	T1	N0	I
17	male	61	low	T1	N0	I
18	male	72	low	T3	N3a	IV
19	male	62	Middle	T3	N3	IV
20	male	57	Middle	T4a	N2	IV
21	male	56	low	T2	N0	IB
22	male	50	low	T4a	N1	IV
23	female	72	low	T2	N0	IB
24	male	77	Middle	T1	N0	IA
25	male	64	Middle	T2	N2	II
26	male	48	low	T4a	N3b	IV
27	female	76	Middle	T2	N0	IB
28	male	62	Middle	T1	N0	I
29	female	74	low	T2	N0	IB
30	female	64	Middle	T2	N1	II
31	male	64	low	T3	N3	IV
32	male	52	Middle	T2	N0	IB
33	female	72	low	T3	N1	III
34	male	60	Middle	T2	N1	IB
35	female	56	low	T3	N3a	IV
36	male	73	low	T3	N1	IV
37	female	59	Middle	T4a	N3	IV
38	female	67	low	T2	N0	IB
39	female	64	low	T2	N0	IA
40	male	66	low	T2	N0	IB
41	female	50	low	T3	N3b	IV
42	male	64	Middle	T3	N2	IV
43	male	58	low	T4a	N2	IV
44	female	66	High	T3	N1	III
45	male	81	low	T3	N1	III
46	male	69	Middle	T3	N2	IV
47	male	47	Middle	T3	N2	IV
48	male	70	Middle	T3	N1	III
49	female	65	High	T2	N1	IB

Supplementary Table 7: The clinical parameters of GC patients and tumors in training set

sample	Gender	Age	Tumor size	Tumor grade	Depth of invasion	LN metastasis	TNM stage
1	male	67	3.0 × 2.0 × 1.0	Middle	T4a	N1	IIIC
2	female	36	1.5 × 1.0 × 1.0	Middle	T4	N2	IV
3	male	65	4.5 × 3.5 × 1.0	low	T2	N2	IIB
4	female	59	1.2 × 1.0 × 0.2	Middle	T1-2	N0	I
5	female	39	4.0 × 3.0 × 1.5	low	T4	N1	IV
6	male	71		Middle			
7	male	58	3.5 × 2.5 × 1.5	Middle	T4	N3	IIIC
8	female	68	5.0 × 5.0 × 1.0	Middle	T2	N0	IB
9	female	55	1.5 × 1.2 × 1.0	High	T1	N0	IA
10	male	71	6.0 × 5.0 × 1.5	Middle	T4b	Nx	IIIC
11	male	60	1.5 × 1.3 × 0.3	Middle	T1	N0	IA
12	male	37		Middle	T2	N0	IV
13	male	73	2.0 × 1.3 × 0.5	Middle	T2b	N0	IB
14	male	75	7.0 × 6.0 × 1.0	Middle	T4	N1	IIIA
15	female	51	4.0 × 3.0 × 0.6	low	T4	N3	IV
16	female	51	4.0 × 3.0 × 2.0	Middle	T4	N1	IIIA
17	male	77	5.0 × 4.0 × 1.0	Middle	T4	NX	IV
18	male	61	0.2 × 0.2 × 0.1	low	T2	N0	IB
19	male	55	3.5 × 2.2 × 1.0	Middle	T4b	N3a	IIIC
20	female	70		low	T4	N2	IV
21	female	43		Middle	T2	N1	II
22	male	65	6.0 × 5.0 × 2.0	low	T4b	N3	IIIC
23	male	64	9.5 × 6.0 × 1.0	Middle	T2	N0	IB
24	male	47	1.6 × 0.3 × 0.2	low	T1a	N0	IA
25	male	37	6.0 × 5.0	low	T4	N1	IV
26	male	58	4.5 × 5.0 × 1.5	High	T4	Nx	IV
27	male	52	4.0 × 3.5 × 1.0	low	T4	N3	IV
28	female	65		low	T3	N2	III
29	female	70		low	T3	N2	III
30	male	68	4.0 × 3.0 × 2.0	low	T1	N0	I
31	male	65	6.0 × 6.0				
32	male	74	7.0 × 6.0	low	T3	N1	IIIA
33	female	62	2.0 × 1.3 × 0.4	Middle	T1	N1	I
34	female	64		Middle	T2	N1	II
35	male	55		low	T3	N1	IIIC
36	male	64		low	T4	Nx	IV
37	male	69	4.0 × 3.5 × 1.5	low	T4	N2	IIIB
38	male	58	6.5 × 3.5 × 0.3	low	T1	N0	IA
39	female	63	8.0 × 1.5	low	T4	N1	III
40	female	54		Middle	T2	N1	II
41	male	69					
42	male	57	8.0 × 6.0 × 1.0	low	T4b	N2	IV
43	male	47		Middle	T3	N2	III
44	male	78	7.0 × 6.0 × 1.5	low	T2	N0	IB
45	male	59	1.5 × 1.3 × 0.4	low	T2	N0	IB
46	male	56		low	T2	N0	I
47	male	45		low	T2	N2	II
48	male	72		low	T4	N2	IV
49	male	86		low	T3	N2	III
50	male	43		Middle	T2	N1	II
51	female	72		low			

Supplementary Table 8: The clinical parameters of GC patients and tumors in testing set

sample	Gender	Age	Tumor size(cm)	Depth of invasion	LN metastasis	Metastasis
1	female	71	3.0 × 3.0 × 1.0	T3	N3	
2	male	66	4.0 × 4.0 × 0.8	T3	N2	
3	male	63	11.0 × 7.0 × 5.5	T3	N0	
4	female	76	3.5 × 3.0 × 0.3	T3	N2	
5	female	69	4.5 × 2.0 × 1.0	T3	N2	
6	female	55	7.8 × 6.0 × 0.8	T3	N0	
7	male	66	10.0 × 7.0 × 3.0	T3	N1	
8	male	56	3.0 × 2.5 × 0.3	T3	N1	
9	male	84	5.3 × 4.3 × 0.3	T2	N1	
10	female	60	4.5 × 3.8 × 0.4	T3	N2	
11	male	61	4.0 × 3.0 × 1.0	T3	N1	
12	female	53	4.0 × 4.0 × 0.8	T3	N2	
13	male	64	3.0 × 2.8 × 0.6	T3	N2	
14	male	58	5.0 × 4.0 × 0.5	T3	N2	
15	male	54	5.0 × 5.0 × 0.5	T2	N0	
16	female	56	5.0 × 4.0 × 1.0	T2	N2	
17	male	56	3.0 × 3.0 × 0.5	N1	N1	
18	male	54	3.0 × 3.0 × 0.2	N3	N3	
19	male	35	6.0 × 4.0 × 1.0	N0	N0	
20	male	74	14.0 × 3.5 × 2.8	N1	N1	
21	male	61	8.0 × 5.5 × 2.0	N2	N2	
22	male	47	2.5 × 2.0 × 0.8	N1	N1	
23	male	51	2.5 × 2.5 × 1.2	N0	N0	
24	male	68	4.0 × 3.0 × 0.4	N1	N1	
25	male	59	3.0 × 2.5 × 0.5	N2	N2	
26	male	75	8.0 × 7.0 × 1.2	N1	N1	
27	male	51	5.5 × 4.5 × 0.8	N3	N3	M1
28	male	40	6.0 × 4.0 × 0.8	N0	N0	
29	female	56	4.0 × 3.0 × 1.2	N3	N3	
30	male	57	4.0 × 3.0 × 1.2	N2	N2	
31	male	66	3.5 × 3.0 × 1.5	N3	N3	
32	male	48		N1	N1	
33	female	48	8.0 × 6.0 × 1.8	N1	N1	
34	male	62	10.0 × 7.0 × 2.0	N2	N2	
35	male	55	2.0 × 1.5 × 0.5	N0	N0	
36	male	52	6.0 × 5.0 × 3.0			
37	male	81	7.0 × 5.0 × 1.5	N0	N0	
38	male	64	3.5 × 2.5 × 0.5	N1	N1	
39	male	59	4.0 × 3.0 × 1.0	N2	N2	
40	female	68	6.5 × 4.0 × 1.0	N3	N3	
41	male	57	5.0 × 4.5 × 0.8	N2	N2	
42	male	67				
43	male	47	3.5 × 3.5 × 2.0	N1	N1	
44	male	69	10.0 × 8.0 × 1.0	N3	N3	
45	male	47	4.0 × 4.0 × 1.5	N2	N2	
46	male	59	5.5 × 5.0 × 2.5	N0	N0	
47	male	53	5.0 × 5.0 × 1.5	N1	N1	

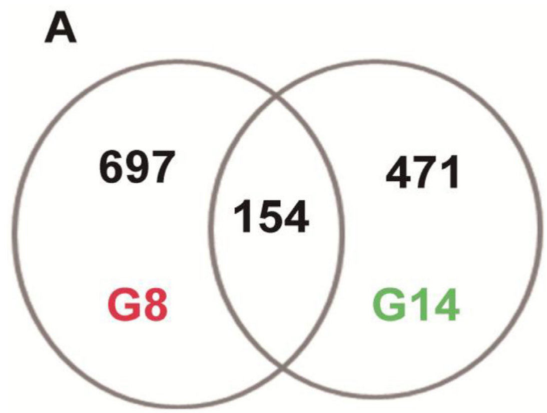

Supplementary Figure 1: Numbers of lncRNAs aberrantly expressed in gastric tissue microarrays. Numbers of aberrantly expressed lncRNAs increased (**A**) and decreased (**B**) in gastric tissue microarrays.

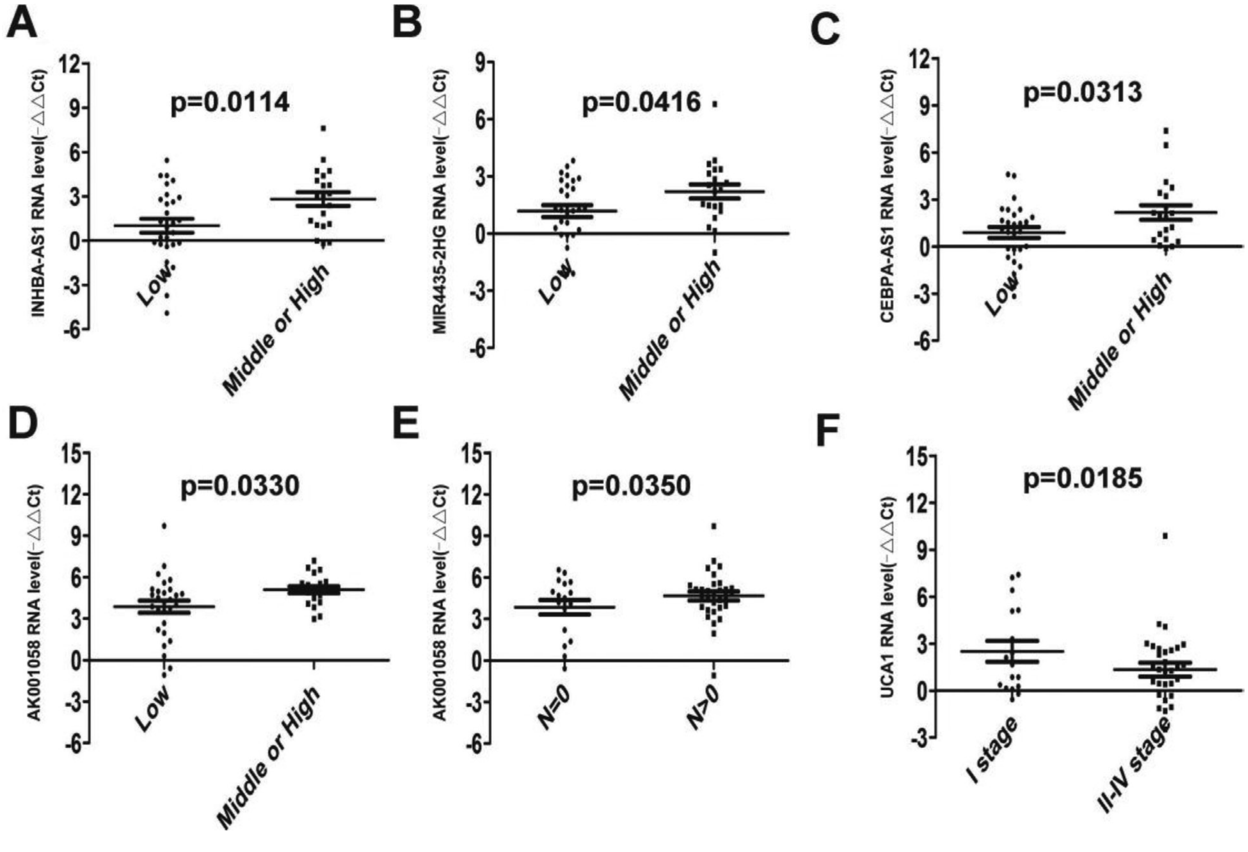

Supplementary Figure 2: The relationship between lncRNA levels in gastric tissues and the clinicopathological features of GC patients. The relationship between the expression level of INHBA-AS1 (**A**), MIR4435-2HG (**B**), CEBPA-AS1 (**C**), and AK001058 (**D**) and tumor grade; The relationship between the expression level of AK001058 and GC lymph node metastasis (**E**); The relationship between the expression level of UCA1 and GC TNM stage (**F**). $-\Delta\Delta Ct = (Ct_{target\ gene\ in\ normal} - Ct_{18S\ rRNA\ in\ normal}) - (Ct_{target\ gene\ in\ cancer} - Ct_{18S\ rRNA\ in\ cancer})$.

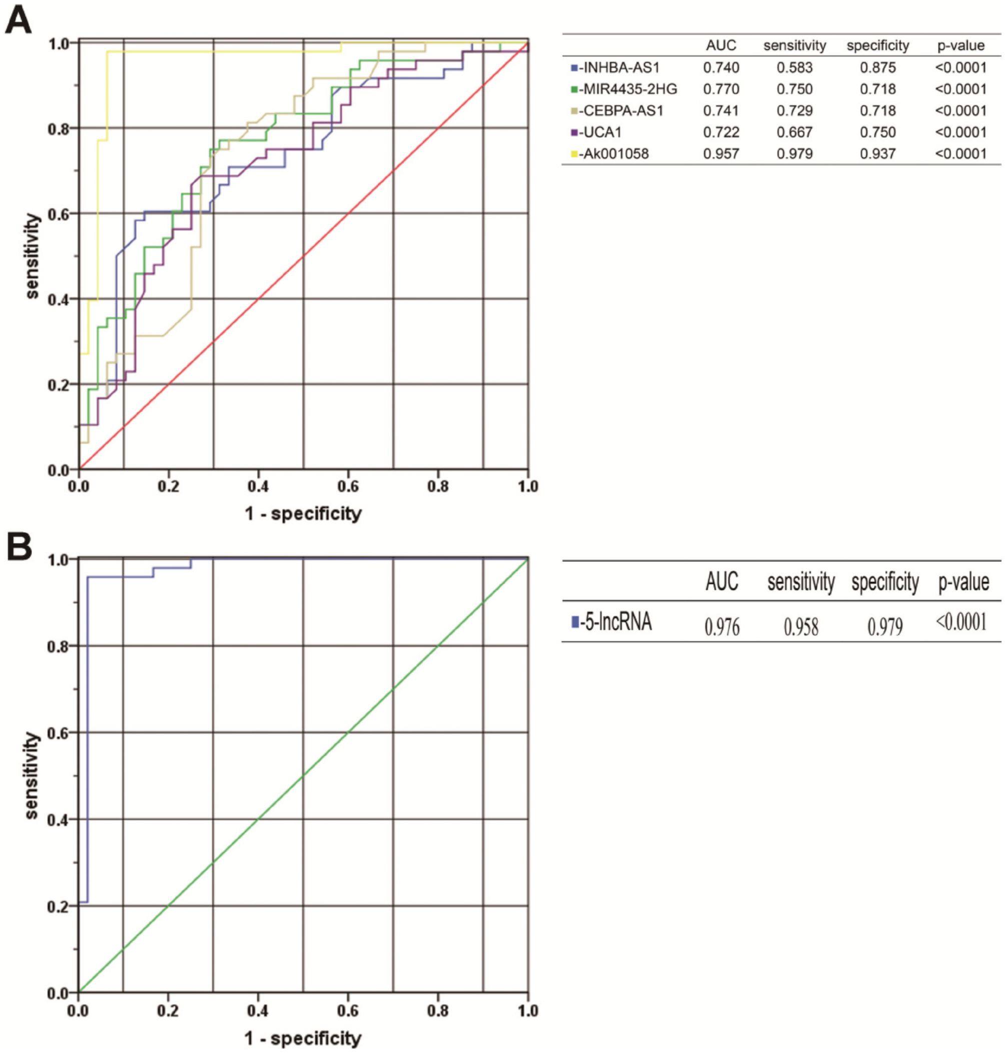

Supplementary Figure 3: AUC for 5 lncRNAs and 5-lncRNA panel in gastric tissues. The ROC curves of the 5 lncRNAs (**A**) and 5-lncRNAs (**B**) expressed in gastric tissues.

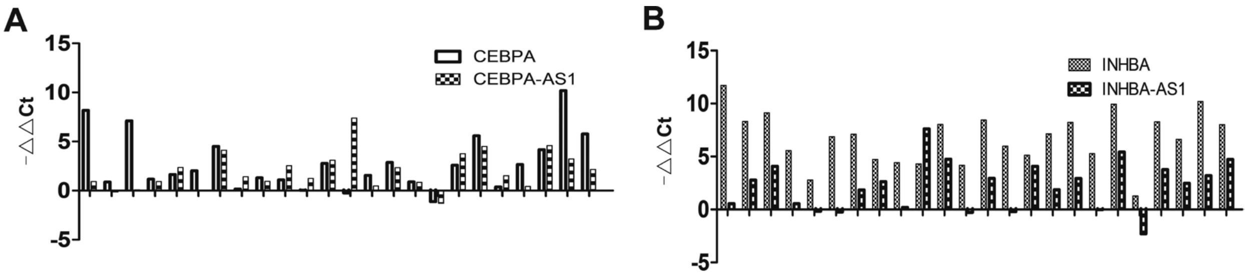

Supplementary Figure 4: The expression levels of lncRNAs and their associated PCGs in gastric tissues. Expression of CEBPA-AS1 (**A**), INHBA-AS1 (**B**) and their associated PCGs in 24 paired gastric tissues, respectively. $-\Delta\Delta Ct = (Ct_{\text{target gene in normal}} - Ct_{\text{18S rRNA in normal}}) - (Ct_{\text{target gene in cancer}} - Ct_{\text{18S rRNA in cancer}})$.
